# Migration of Volatile Organic Compounds (VOCs) from PEX-a Pipes into the Drinking Water during the First Five Years of Use

**DOI:** 10.3390/ma14040746

**Published:** 2021-02-05

**Authors:** Aino Pelto-Huikko, Merja Ahonen, Mia Ruismäki, Tuija Kaunisto, Martti Latva

**Affiliations:** 1 WANDER Nordic Water and Materials Institute, Satakunta University of Applied Sciences, PL 211, FI-26100 Rauma, Finland; merja.ahonen@samk.fi (M.A.); tuija.kaunisto@samk.fi (T.K.); martti.latva@samk.fi (M.L.); 2 Clean Technology, Metropolia University of Applied Sciences, Leiritie 1, FI-01600 Vantaa, Finland; mia.ruismaki@metropolia.fi

**Keywords:** PEX-a pipes, plastic materials, corrosion, drinking water, tert-butyl alcohol

## Abstract

A brand-new office building in Rauma, Finland, was used to study the first five years of PEX-a drinking water pipes in normal use. Both pipe material and water samples from hot and cold-water pipelines were analyzed. Migration of volatile organic compounds (VOC) from the PEX-a pipes into the drinking water was observed to decrease rapidly during the first months. Deterioration of the PEX-a material was observed to take place due to the wearing down of organic antioxidants added into the PEX-a material during the manufacturing of the pipes. Tert-butyl alcohol (TBA) concentrations were high during the first months after commissioning of use. The stagnation time of the drinking water in contact with the PEX-a material before the actual water sample was taken had a major impact on analyzed migration of organic compounds. Hence, the amount of organic compounds able to migrate from materials into the drinking water will increase when the stagnation time increases. In this study, the water samples were taken after overnight stagnation, whereas in normal use it is advisable to run water properly before drinking it. Instructions will be needed for the average user to avoid harmful health effects.

## 1. Introduction

The most typical materials in installed drinking water systems inside buildings are stainless steel, carbon steel, copper, and polyethylene. Polyethylene pipes are generally either polyethylene, cross-linked polyethylene (PEX), or multilayer pipes. They have many useful features, such as long durability, corrosion resistance, low cost, and easy installation. Plastic materials used in water systems do not experience traditional corrosion, but their ageing processes have impact on durability of the pipes. In practice, physical deterioration, oxidation, hydrolyzing, thermal decomposition, and combination of these may occur. Small differences in raw-material content or in manufacturing processes may have significant effect on to the properties of the plastic in the long run.

For example, increasingly being used in drinking water systems, pipes made of polyethylene undergo degradation, e.g., due to disinfectants in the water. Chlorine (Cl_2_) and chlorine dioxide (ClO_2_) are the most common water treatment chemicals used for disinfection and they are strong oxidants even at a very low concentration. There are several studies concerning the deterioration of polyethylene pipes with waters containing chlorine [1,2,3,4,5,6,7].

In addition, studies exists on the possible health effects of chemicals migrated to drinking water when using plastic drinking water pipes [8,9,10,11]. However, as there will be more and more new installations where plastic materials are utilized, it is necessary to be aware of the amounts of different compounds that may be released into the drinking water from plastic pipes. In fact, the critical concern facing PEX-pipes is the potential leakage of additives, for example antioxidants, stabilizers, adhesives, and breakdown products thereof into water in contact with pipe surface [12,13,14]. 

The identity of the chemicals leaking from PEX-pipes is linked to their manufacturing method. Cross-linking of polyethylene in PEX-pipes is achieved by three main processes: using peroxide (PEX-a), using the ‘silane’ method (PEX-b), or using irradiation (PEX-c). In manufacturing plastic pipes, a number of different chemicals are used in addition to a base polymer to improve the properties of the final product. 

Polyethylene is the main raw material in drinking water systems inside buildings, which itself does not dissolve in water. However, Nielsen and co-authors have observed that antioxidants dissolved from one and three year old polyethylene pipes produced by different manufacturers [15]. More antioxidants were observed to dissolve from new pipes. Additionally, crosslinked PEX pipes manufactured using different technologies and by different manufacturers have been found to release small amounts of antioxidants [14]. For example, Whelton and Nuygen found in their water samples studies degraded products of substances like methyl tert-butyl ether (MTBE) and ethyl tertiary butyl ether (ETBE), which are used as initiators in the cross-linking process of polyethylene [16]. In the same study, they observed in water samples decomposition products from chemicals used in the manufacturing of plastic pipes like antioxidants, such as 2,4, di-tert-butylphenol and 2,6-di-tert-p-benzoquinone, and an antioxidant, 4-methyl-2,6-di-tert-butylphenol, as well as 5-methyl-2-hexanone, which is a decomposition product of polyethylene. Additionally, earlier Brocca et al. discovered traces of antioxidants and their degradation products such as 3,5-di-tert-butyl-4-hydroxy-benzaldehyde and 7,9-di-tert-butyl-1-oxaspiro[4,5]-deca-6,9-diene-2,8-dione [13].

Migration of additives have influence on the ageing of polyethylenes, but there is not much information related to their long-term effects. Aggressive substances impact on the ageing of polyethylene materials. Especially in a situation where the operating temperature is higher than specified by the manufacturer, the risk of failure is higher [17].

In this study, new information has been obtained on soluble compounds of PEX-a pipes and on their dissolution rate with respect to the time that pipes are used. This information will be needed in EU countries in setting product approval requirements for plastic products and materials in contact with drinking water. For organic compounds in some countries, such as Germany and the Netherlands, there exists a positive list of the chemicals allowed to use in products targeted for use in drinking water systems. In some EU countries, health authorities are currently investigating setting of limits for certain undesirable compounds released from plastic pipes.

The office building Technology Centre Sytytin in Rauma, Finland was used as a real-life research environment in this project. The research was focused on the first five years of use after commissioning. Samples of the plastic drinking water pipe material and water samples were studied from hot and cold-water pipelines. Previously, studies related to the microbiological growth inside the pipes, the impact of magnetic water treatment and migration of metallic materials from copper pipes and brass components utilizing this unique real-life water system, have been reported [18,19,20].

## 2. Materials and Methods

### 2.1. Water System in the Office Building

**Figure 1:**
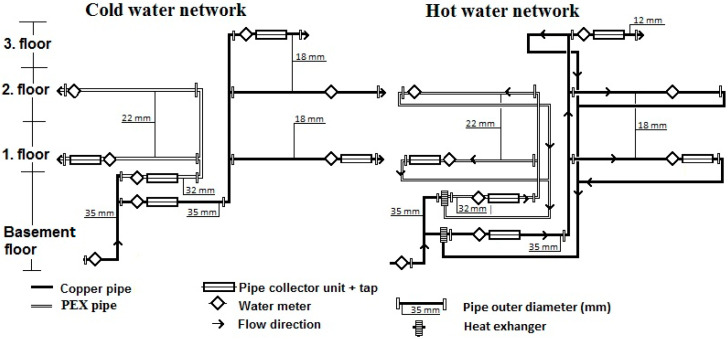
Schematic drawing of the structure of the water systems in office building.

As part of a larger long-term research program related to the interactions between drinking water and materials this study focused on water supply pipes made from PEX-a material. The study composed of two parts. The first part was focused on studying water samples collected from both hot (hot-water average temperature 55.0 ± 1.0 °C) and cold-water pipes (cold-water average temperature 16.0 ± 4.5 °C). The second part focused on pipe material samples from the same hot and cold-water pipes after five years of use. This research was carried out in the actual cold and hot-water systems which were in everyday use by the people working in the office building. In the Figure 1, the schematic structure of the studied water system is shown. Both copper pipes and PEX-a pipes represent typical pipes of a quality manufactured, sold and used inside the EU. The copper pipes manufactured according to the standard EN 1057 and PEX-a pipes according to the EN ISO 15875 were acquired from Onninen Ltd, Finland.

The study was performed in an office building where 250 employees work with regular office hours, from Monday to Friday 8 am to 4 pm, situated in Rauma, Finland. The water distribution system of the building was designed for full-scale research purposes and had been planned and installed according to the current legislation and protocols in Finland. Before the pipe network commissioning, all pipes were rinsed with tap water to remove any possible dirt and loose materials. Water sampling for this study was started immediately after commissioning, on the first working day. The full-scale water distribution network contained detachable pipe specimens at 11 various points inside the building. The pipe specimen is a short piece of pipe connected with brass couplings to the water system. In order to study the materials, the pipe specimen with couplings was detached from the system and replaced with a new specimen and couplings. At each 11 points, five pipe specimens have been installed in a row (pipe collector unit) to enable long term research. 

The water distributed to the research site by the Rauma waterworks was purified from surface water. The water purification was a two-phase process with the first phase consisting of vertical sedimentation at pH 5 with ferric sulfate as the precipitation chemical and Ca(OH)_2_ as the pH control chemical. The second phase included flotation with the same chemicals as used in the vertical sedimentation. Disinfection and manganese oxidation were performed using sodium hypochlorite and active carbon filtration was performed to complete the treatment. Ammonium chloride was fed into the distribution network to guarantee chlorine stability throughout the whole distribution network. The waterworks where water treatment took place is located 4.4 km from the office building.

### 2.2. Pilot-Scale Water System

In addition, a pilot scale water distribution system built in the laboratory was utilized in this research. The structure of the pilot scale distribution system has been described earlier [19]. The pilot scale network encompasses eight parallel lines of looping pipes that are mounted on the laboratory walls. Overall, four of the lines were constructed of 11 m long high-density cross-linked polyethylene (PEX-a) pipes and at the end of each line there was a tap for water sampling. The quality and producer of PEX-a material is the same as is in the water system of the building.

### 2.3. Water Analysis

In total, 100 mL water samples were collected after overnight stagnation from the water system in the office building. Collection was undertaken from the point before water entered the distribution network of the building, as well as from the sampling points before and after each pipe collector located either on basement floor, first floor or third floor. To study the impact of stagnation time of water on measured amounts of migrated organic compounds, a pilot scale water system located in the first-floor laboratory was utilized. Here also, 100 mL water samples were collected after different stagnation times. Temperatures of the water samples were measured on a calibrated digital thermometer with stainless steel stem purchased from VWR International (Radnor, PA, USA).

The water analyses were performed using a gas chromatography–mass spectrometer (GC–MS) (QP2010 Ultra, Shimadzu, Kyoto, Japan). Head-space injection was used in analyzing volatile organic compounds (VOC) and liquid injection in analyzing semi volatile organic compounds (SVOC). SVOC samples were prepared with liquid-liquid extraction with dichloromethane as the solvent. The extraction process and concentrating were optimized by experimenting with different extractions and concentrations. A functioning method was obtained for VOCs where the different compounds could be separated and analyzed quantitatively from water samples. 

### 2.4. Materials Analysis

Pipe samples were removed from the pipe collector systems after 5 years of use. From these material samples oxidation induction times (OIT) were measured. FTIR-analysis and high-pressure liquid chromatography (HPLC) analysis were also performed.

High-performance pressure liquid chromatography analyses were done using Agilent 1200 chromatograph (Palo Alto, CA, USA) equipped with the UV-detector. Wavelength of the detection was 276 nm. Sample was prepared by diluting the sample in organic solvent. In the chromatograph column C-18 was used.

Fourier Transformed Infra-Red (FTIR) Spectroscopy analyses were done on a Perkin Elmer spectrum GX Fourier Transform Infrared Spectrometer (Perkin Elmer, Waltham, MA, USA) performing surface analysis (ATR = Attenuated Total Reflectance (single reflection)) 4000–650 cm^−1^.

Determination of oxidation induction time (OIT) was done using Differential Scanning Calorimetry. The OIT measured the baseline of the oxidative-reaction intercept in the extrapolated tangent to the exothermic reaction. Netzsch 204-F1 (Netzsch, Selb, Germany) differential scanning calorimeter was used. The procedure used was: 1. heating from 30 °C to 210 °C in N_2_ (99,999%) atmosphere with heating rate 20 °C/min, 2. isothermal hold at 210 °C for 3 min in N_2_ atmosphere, 3. switch to purge of Oxygen gas (99,999%) with flow rate at 50 mL/min, 4. isothermal hold until oxidation completed. Exposure was performed in open aluminum crucibles. The three parallel samples were analyzed. 

## 3. Results and Discussion

### 3.1. Water Quality Studies

Water samples were taken regularly during the first five years of use of the office building. Samples representing seven different sampling times covering the whole five year period were analyzed in this study. 

**Figure 2:**
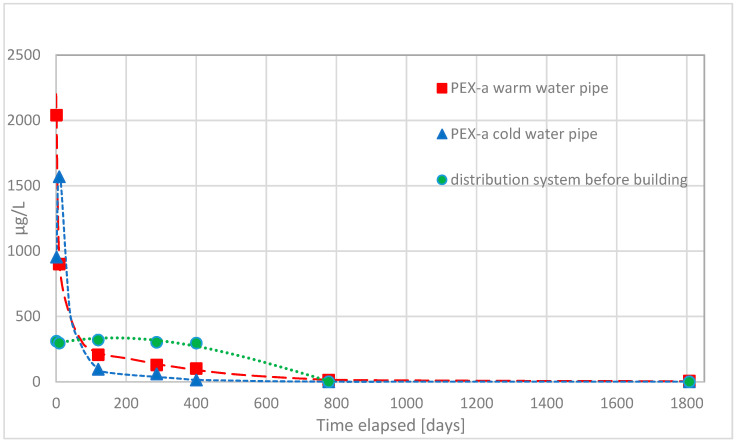
The observed concentrations of migrated tert-butyl alcohol (TBA), during the first five years of use of the office building, from the PEX-a water pipes into the drinking water and their corresponding concentration in the water entering the building from the waterworks distribution system.

**Figure 3:**
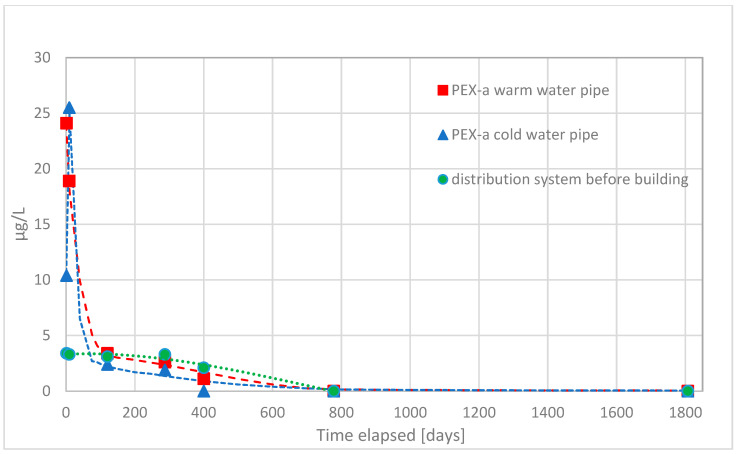
The observed concentrations of migrated methyl tert-butyl ether (MTBE) during the first five years of use of the office building, from the PEX-a water pipes into the drinking water in their corresponding concentration in the water entering the building from the waterworks distribution system.

**Figure 4:**
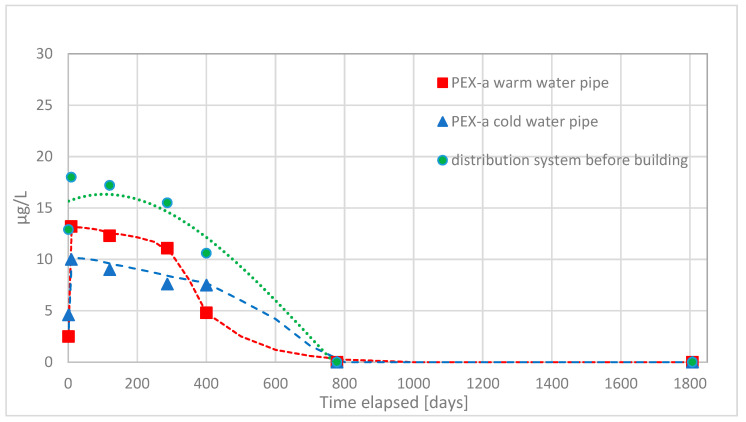
The observed concentrations of migrated ethyl tert-butyl ether (ETBE) during the first five years of use of the office building, from the PEX-a water pipes into the drinking water and their corresponding concentration in the water entering the building from the waterworks distribution system.

**Figure 5:**
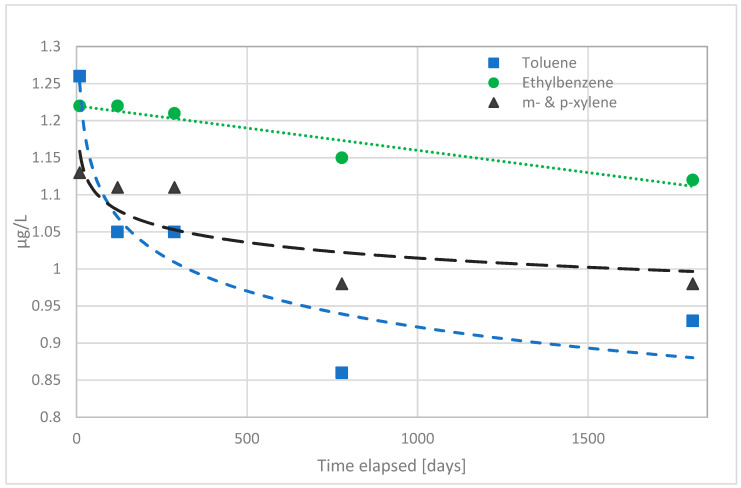
The measured concentrations of ethylbenzene, m-xylene and p-xylene in the water entering the office building during the first five years use.

First, volatile organic compounds were analyzed from water samples. Water samples were found to contain moderate amounts of methyl tert-butyl ether (MTBE), ethyl tert-butyl ether (ETBE), and tert-butyl alcohol (TBA) originating from compounds used to initiate crosslinking reaction of PEX-a. Of these, the first two produce odor and taste disturbances in water, and the third, tert-butyl alcohol, is known to cause damages to the kidneys [10]. In the Figure 2, Figure 3, Figure 4 and Figure 5 concentrations of these substances as a function of time in both hot and cold water are shown. 

In addition, the Figures show values for water entering the building and surprisingly some amounts of the VOC compounds are already present in the water. Most probably they originate from the materials in the waterworks network before drinking water enters the building. Tert-amyl methyl ether and benzene were not found. The amounts of ethylbenzene, toluene, p-xylene and m-xylene did not increase in the building network. Most likely these materials have not migrated from the water system materials inside the building, but from the materials outside the building belonging to the waterworks distribution system. During the construction phase of the office building, Technology Centre Sytytin, a new 100 m long service line produced from non-crosslinked polyethylene was installed. Most likely this new underground pipeline is responsible of the migration of several organic compounds, which are seen here as elevated background values measured from the sampling point located before the drinking water encounters the PEX-a pipe materials of the building.

TBA and MTBE migration behavior was equal. Migration into the drinking water was highest right after commissioning. A slight increase was seen during the first two days before the actual continuous decrease starts. The strongest decrease in migration of TBA and MTBE lasts circa 100 days, i.e., three months. After that migration continued to decrease continuously until after circa 800 days no TBA and MTBE migrated anymore. There was not much difference between warm and cold water except slightly more TBA and MTBE migrated into the warm water.

The migration of ETBE from PEX-a pipes was much less than for TBA and MTBE. The decrease in migration as a function of time used was not so strong right after commissioning and migration continued almost at the same level circa 300 days for warm water and 400 days for cold water. The migration of ETBE into the drinking water was clearly less and slower than the migration of TBA and MTBE. 

It was also clear that migration of TBA, MTBE, and ETBE into the drinking water takes place most likely from the service pipe installed before the building. Migration from the service pipe continued for those longer time periods most likely because of the length of the pipe and lower temperature of water.

In addition, toluene, ethylbenzene and m- and p-xylene were found in the drinking water, but their amounts did not increase due to the pipelines inside building. Those were present already in the incoming water. Besides VOCs, other organic compounds were studied from the water samples using GC–MS method. However, only small amounts of 2,4-Di-tert-butyphenol, 3,5-Di-tert-butyl-4-hydroxybenzaldehyde, 7,9-Di-tert-butyl-1-oxaspiro[4,5]-deca-6,9-diene-2,8-dione, Mesityl oxide, and Styrene were observed from hot-water samples. From cold water samples, the same compounds except 3,5-Di-tert-butyl-4-hydroxybenzaldehyde, and 7,9-Di-tert-butyl-1-oxaspiro[4,5]-deca-6,9-diene-2,8-dione were found. No clear changes during the time elapsed were noticed for any of these. However, one unidentified high molecular weight compound was also found from hot-water samples and the concentration of it decreased in the same manner as e.g., for TBA as the pipes were used.

When water samples were analyzed the most interesting question was how long water stayed in contact with PEX-a material before a water sample was taken. The impact of stagnation time of water inside PEX-a pipe on measured values was studied using cold-water pipes of the pilot scale distribution system.

Because there were multiple users and the building was in an actual working environment, it was quite difficult to take water samples after longer stagnation times and control exact stagnation times. For this reason, instead of using the real-life water system, the impact of stagnation time of water inside PEX-a pipe on measured values was studied using cold-water pipes of the pilot scale distribution system. The six different stagnation times between 0 h (as control) and 24 h were studied. Stagnation time 24 h was chosen to be the longest time while five other stagnation times were chosen to represent evenly the time frame from 0 to 24 h. To minimize the impact of total time of use, pipes had been in use for 6 months before stagnation time studies. Hence, it was possible to take samples from the same pipeline, at different stagnation times, in a way that all different stagnation times we studied separately and successively. This experiment was not possible to be repeated equally because the initial situation of the pipe had already changed due to the first experiment.

**Figure 6:**
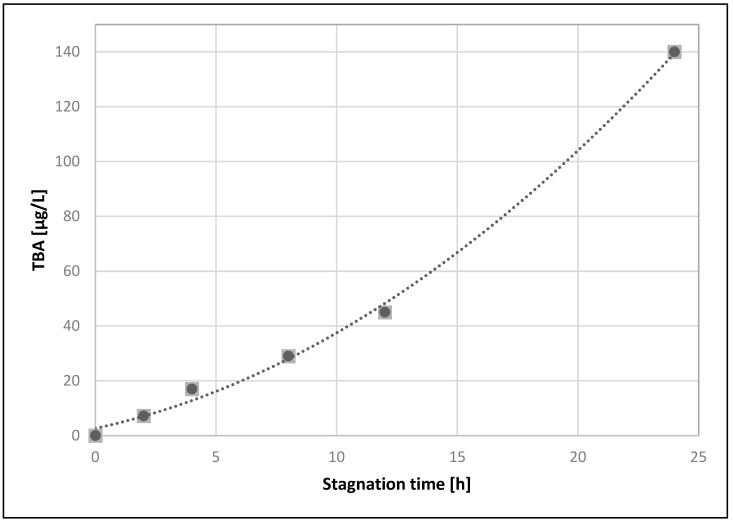
The amount of migration of tert-butyl alcohol into drinking water as a function of stagnation time.

Only TBA was monitored from the water samples. The results are shown in Figure 6, where the amount of migrated TBA into the drinking water as a function of stagnation time is presented. The Figure shows that the amount of migrated TBA increases as stagnation time increases from 0 to 24 h. Correlation slightly deviates from the linear response and a second order polynomial function can be fitted clearly better. This is most likely because the stagnation time also affects temperature of the water. Temperature of the water increases closer to the indoor temperature when the water is stagnated inside the water system of the building. This change of temperature towards the typical indoor environment temperatures occurs despite the cold-water pipes being insulated. Higher temperatures typically increase the migration of organic substances from plastic materials into drinking water.

### 3.2. Pipe Material Studies

Material studies were performed on pipe samples taken after five years of use. Changes in the materials were studied utilizing three different techniques, i.e., FTIR spectroscopy, high performance liquid chromatography (HPLC), and calorimetry, where the time between melting of the material and its actual decomposition is measured (oxidation induction time, OIT). HPLC studies were used to determine the levels of antioxidants remaining in the PEX-a pipes. In the PEX-a pipes analyzed in these studies only one antioxidant, octadecyl-3-(3’,5’-di-tert-butyl-4-hydroxyphenyl) propionate (AO1076) was used. AO1076 is a long-term heat stabilizer for polymer material. The measured amount of it in the original unused pipe was 1920 ppm. In the basement floor pipe sample, the amount of antioxidant in the hot water pipe (D = 32 mm) decreased to 350 ppm and in the first-floor pipe sample (D = 22 mm) to 860 ppm after 5 years of use. Similarly, in the cold-water pipes, the basement floor pipe sample had an antioxidant content of 1440 ppm and in the first-floor pipe sample the content was 1510 ppm. The amount of the antioxidant in the hot-water PEX-a pipes has decreased clearly from the original level and was also clearly lower than in the corresponding cold-water pipe samples. Hot water clearly consumes more antioxidant than cold water because organic compounds are usually more soluble in hot water. In addition, more water circulates in the hot-water pipes than in the cold-water pipes because the office building has a continuous hot-water circulation. Due to the higher amount of water flowing through the pipes in the basement floor, the amounts of antioxidant in these pipe samples were reduced for both the cold and hot-water pipes, more than for the pipe samples from the first floor.

FTIR spectroscopy was utilized to investigate the degradation of the polyethylene polymer. Decomposition takes place when the typical carbon-carbon bond in the polymer is broken-up and oxidized to carbonyl. Consequently a C=O double bond will be detected in the IR-spectrum. The formation of carbonyl indicating polymer degradation occurs in the spectrum at the wavelength range 1710–1715 cm^−1^. However, none of the samples studied showed any peak at the respective wavelengths, which would have indicated the breakup and oxidation of the polymer. The result is consistent with HPLC results in that the amount of antioxidant was still sufficient to prevent oxidation of the actual polymeric material. However, it was noticeable on the IR-spectra that the formation of carbonyl from the tubes studied will most likely first occur in the hot-water pipes in the basement floor, where also the number of antioxidants has decreased the most.

**Figure 7:**
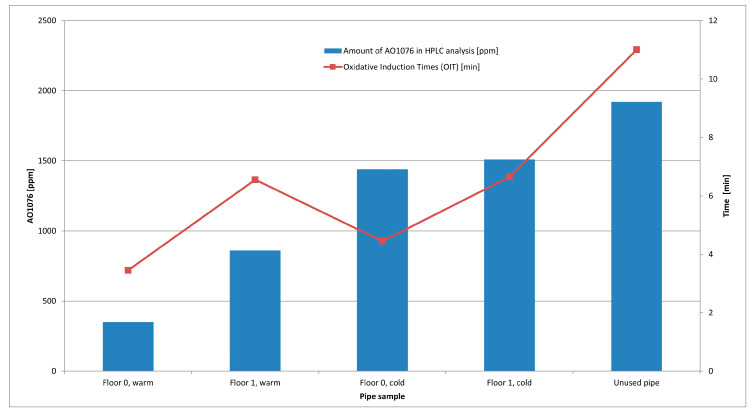
The Oxidative Induction Times (OIT) of the examined plastic pipe samples and the quantities (ppm) of antioxidant determined AO1076 by HPLC.

In Oxidative Induction Times (OIT) testing using Differential Scanning Calorimetry, it was found that the average time required for starting the decomposition of the material was 11.0 min for the unused PEX-a pipe. The corresponding times for the samples from the pipes used for five years were shorter than that. The time required to start the decomposition of hot-water pipe samples in the OIT-testing was 3.45 min for the basement floor pipe sample and 6.55 min for the first-floor pipe sample. Similarly, OIT times for cold-water samples were 4.45 min for the basement floor pipe sample and 6.65 min for the first-floor pipe sample. The results were consistent with the HPLC measurements except for the cold-water pipe sample from the basement floor. Despite of the fact that, based on the HPLC analysis, there was found clearly more antioxidant AO1076 left in the basement-floor cold-water pipe sample than in the first-floor hot-water pipe sample, the OIT measurements made a clear distinction showing higher OIT time for first floor hot-water pipe than for cold-water pipe from basement-floor. The OIT and HPLC results are shown in the Figure 7.

## 4. Conclusions

Corrosion occurred after five years of use in some of the brass couplings in Technology Center Sytytin. This was due to the dezincification of brass, which is not dezincification resistant. This especially occurred in the early stages of use, and resulted in high amounts of lead, copper, and zinc being dissolved into the drinking water. Of these, especially copper ions are known to have a slight effect on the durability of plastic polymers [21]. However, it is impossible to estimate how much impact it has on decreasing the antioxidant concentrations in the studied PEX-a pipe samples.

Based on studies with water samples, the high TBA concentrations measured in the early stage of use will need instructions for the average users to avoid harmful health effects. MTBE and ETBE remained at a fairly reasonable level already in an early stage of use. Of these three compounds TBA is likely to be the most potential to possess health risks and will need a health-based limit value when evaluating the quality of drinking water.

The stagnation time of drinking water in contact with materials of the water system, before the actual water sample is taken, is an important factor when evaluating the results. The amounts of dissolved organics will increase when the stagnation time increases for organic pipe materials in contact with drinking water, as is also the case with the release of metal ions from metallic materials in contact with drinking water. In this study, the water samples were taken after overnight stagnation showing increased amounts of migrated compounds in the morning. In normal use it is recommended to run water properly before drinking.

The stagnation time also has effect on the temperature of the water sample. When there was no stagnation the temperature of the water was 13.5 °C but already after 2 h of stagnation the temperature of the water sample was the same as room temperature at 22 °C. It is probable that during stagnation the rise in temperature increases the migration of VOCs.

It should be noted that some of these organic compounds were already found in the water entering the building before the water came into contact with PEX-a material. Therefore, the safety of materials used in waterworks and in their networks should also be studied. Usually, drinking water is in contact for longer periods with those materials than pipes inside buildings. Additionally, prevailing temperatures are typically lower in outdoor underground installations.

Based on the pipe samples, certain amounts of antioxidants from the pipe materials have been consumed during the first five years, but all pipes studied were fairly well-maintained. The consumption of antioxidants is influenced by the water temperature, water quality, size of the pipe, flow speed, and the total amount of water flowing through the pipe.

There is a need for more studies related to the use of plastic drinking water pipes. To evaluate the health effects, toxicological information of several organic compounds that can migrate from plastic materials into drinking water is needed. For regular monitoring of drinking water quality, analysis for organics other than VOCs are still quite laborious and expensive. In addition, possible observations of unidentified compounds must also be solved. Those might be, e.g., decomposing products of additives used in the manufacturing processes of plastic pipes.

## Data Availability

Data Sharing is not applicable.
